# Boosting Depth-Based Face Recognition from a Quality Perspective

**DOI:** 10.3390/s19194124

**Published:** 2019-09-23

**Authors:** Zhenguo Hu, Penghui Gui, Ziqing Feng, Qijun Zhao, Keren Fu, Feng Liu, Zhengxi Liu

**Affiliations:** 1 College of Computer Science, Sichuan University, No.24 South Section 1, Yihuan Road, Chengdu 610065, China; 2017223040014@stu.scu.edu.cn (Z.H.); penghuigui@stu.scu.edu.cn (P.G.); 2017226040002@stu.scu.edu.cn (Z.F.); qjzhao@scu.edu.cn (Q.Z.); liuzhengxi@scu.edu.cn (Z.L.); 2 College of Computer Science and Software Engineering, Shenzhen University, Xueyuan avenue, nanshan district, Shenzhen 518060, China; feng.liu@szu.edu.cn

**Keywords:** depth-based face recognition, deep models, data quality, database

## Abstract

Face recognition using depth data has attracted increasing attention from both academia and industry in the past five years. Previous works show a huge performance gap between high-quality and low-quality depth data. Due to the lack of databases and reasonable evaluations on data quality, very few researchers have focused on boosting depth-based face recognition by enhancing data quality or feature representation. In the paper, we carefully collect a new database including high-quality 3D shapes, low-quality depth images and the corresponding color images of the faces of 902 subjects, which have long been missing in the area. With the database, we make a standard evaluation protocol and propose three strategies to train low-quality depth-based face recognition models with the help of high-quality depth data. Our training strategies could serve as baselines for future research, and their feasibility of boosting low-quality depth-based face recognition is validated by extensive experiments.

## 1. Introduction

Three-dimensional (3D) face recognition (FR) has been studied for several decades with a wide variety of methods proposed [1,2,3,4,5]. It is believed that 3D face data have intrinsic advantages over 2D face images in detecting presentation attacks and in providing additional discriminative features for FR [6,7]. Yet, 3D FR had not gained popularity in real-world applications until Apple Inc. released its iPhone X [8] with TrueDepth camera and Face ID in 2017. One reason is due to the fact that the scanners used for acquiring 3D face in previous studies are often bulky and expensive, so they are thus not feasible in practical scenarios, though previous studies [1,2,3] obtained very high recognition accuracy by using the captured high-quality 3D face data (see Table 1). Here we categorize these methods into high-quality depth-based FR.

**Figure 1:**
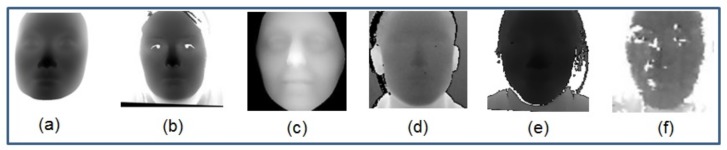
Depth images captured by different devices or under different conditions show different quality levels. From left to right: (**a**) Facial depth images captured by SCU 3D scanner [17] (in our collected database), (**b**) Konica Minolta Vivid 910 [18] and (**c**) 3dMD [19] in lab, (**d**) Kinect II in lab [20], (**e**) RealSense in lab (in our collected database) and (**f**) in the wild [14].

The emergence of low-cost RGB-D sensors, such as Kinect [9] and RealSense [10], makes it possible to capture 3D faces more efficiently and more cost-effectively. Many attempts [11,12,13,14,15,16] have been made in recent years to develop practical FR systems based on RGB-D sensors. As shown in Table 1, in some RGB-D FR scenarios, with depth images as auxiliary information, researchers [13,15] show that FR accuracy can be improved compared with using only RGB images. However, the accuracy achieved by using depth images captured by low-cost RGB-D sensors [11,12,13,15] is still much lower than that by using 3D faces captured by 3D scanners [1,3]. This should be attributed to that the quality of the depth images captured by low-cost RGB-D sensors is generally poor, and we call such data as low-quality depth data (see Figure 1). In contrast to the aforementioned high-quality depth-based FR, we categorized these methods into low-quality depth-based FR. In Figure 1, depth images captured by different sensors are shown, which naturally cause the difference between two kinds of data on resolution and precision. Here, resolution (also known as density) refers to the density of the 3D face point clouds, defined by the number of points used to represent the 3D faces. Higher resolutions mean more details captured on the 3D faces. Precision refers to the minimum measurement error of depth values in unit of millimeters. Thus, smaller values indicate higher precisions.

In the past, regarding low-quality depth-based FR, it was usually an auxiliary of 2D FR. Most researchers focused on how to design a feature extractor or network to gain discriminative feature different from the color images, while very few works care about how the data quality and feature representation of such low-quality data can be enhanced for improving FR accuracy. This is due to two reasons: (1) A database containing both high- and low-quality depth data is lacked; (2) A reasonable and quantitative evaluation on how depth data quality influence the FR performance is underexplored. Please note that essentially, the former will astrict the latter. Therefore, the purpose of our work is to solve the two problems and then propose strategies to boost the performance of the low-quality depth-based FR by improving data quality and feature representation.

In the paper, to solve the data limitation, we extend our Multi-Dim [21] to a large-scale face database called Extended-Multi-Dim database, which consists of: (1) Subjects’ color images, (2) the corresponding low-quality depth images captured by RealSense, and (3) the corresponding high-quality 3D shapes captured by a 3D scanner. The data is captured under varying pose, illumination and expression. We believe that the advent of such a database could boost the research on not only depth-based FR but also other face-related tasks including RGB-D face recognition, 3D face reconstruction and so on. The details about the database will be introduced in Section 3: Extended-Multi-Dim.

Before this work, we did a related evaluation work, which was accepted on CVPR 2019 Biometrics Workshop [22]. In [22], we delved into how depth data quality influences depth-based face recognition and especially two aspects are focused on: precision and resolution. We conducted evaluation on generated high-quality depth images from existing datasets including FRGC V2 [18], BU3D-FE [19], Lock3DFace [20], RGBD-W [14], as well as part data of the Extended-Multi-Dim database which we introduce in this paper. Several significant observations were obtained in [22], demonstrating that precision and resolution are indeed two important factors influencing the recognition accuracy of depth-based FR. In contrast, motivated by the observations in [22], this paper further investigates how to improve the quality of low-quality depth data and identity feature representation with the assistance of high-quality data.

As previously mentioned, with the extended database and the activation of reasonable evaluation, we can focus on how to improve the quality of low-quality depth data, which should cause an improvement on performance of depth-based FR. Here, rather than enhancing data quality through data preprocessing as in [23,24], we expect to extract more discriminative identity feature from low-quality depth face images with the models which are guided by some constraints from high-quality depth data in training phase. This is because the former enhances data quality visually without definitely preserving necessary identity information. In contrast, we focus on how to use the guidance of high-quality data to train a better model for low-quality depth-based FR. In this paper, three strategies are proposed where the high-quality depth data participants and guides the training of low-quality depth-based FR models: image-based strategy, feature-based strategy and fusion of the former two.

The image-based strategy can be formulated as Equation (1), where $x_{l}$ and $x_{h}$ represent the pairs of low and high-quality depth images of the same person, $E_{l}\left(\cdot \right)$ represents a low-quality depth-based extractor, G(·) represents the image generator whose input and output are identity feature of a low-quality image and a produced high-quality image, F(·) is an extractor for generated or true high-quality image. In this scheme, it is the high-quality data images that guide the low-quality depth-based models training. The Equation (2) can formulate the feature-based strategy, where $E_{h}\left(\cdot \right)$ represents an identity feature extractor for high-quality depth images, and the meanings of the other indicators are the same with the Equation (1). In this scheme, it is the identity feature of high-quality data images that guides the models training. Finally, the fusion strategy means that both high-quality depth image and its corresponding identity features guide to train a low-quality depth-based FR model. The specific proposed methods of the three strategies will be introduced in Section 4.

(1) $$F\left(G\left(E_{l}\left(x_{l}\right)\right)\right)\approx F\left(x_{h}\right)$$

(2) $$F\left(E_{l}\left(x_{l}\right)\right)\approx F\left(E_{h}\left(x_{h}\right)\right)$$

To sum up, the contributions of this paper are summarized as follows:

(1) We present a large-scale and multi-modality database Extended-Multi-Dim for FR. It has 902 objects which is the largest public RGB-D database, with the high-quality 3D depth data.
(2) We adopt a series of preprocessing methods for the collected databases including labeling 51 landmarks of 3D shapes and labeling 5 landmarks for RGB-D images.
(3) We design a standard experimental protocol for the collected database. Motivated by some conclusions of previous evaluation, we propose some methods based on three strategies to use the information of high-quality depth data to train a better network for low-quality depth-based FR. The results can be as the benchmarks for other researchers.

The rest of this paper is organized as follows. Section 2 introduces some related works including some public databases and approaches. Section 3 introduces in detail the Extended-Multi-Dim database. Section 4 presents the details of the proposed methods based on three strategies. Section 5 shows the experimental results of the approaches and corresponding analysis about low-quality depth-based FR. Section 6 will conclude the work.

## 2. Related Works

### 2.1. Databases

There are no large-scale public database containing both high- and low-quality face depth data of each object. The databases consisting of depth data are usually used for high-quality depth-based FR or RGB-D FR, which can only capture one kind of depth data.

The common databases used in high-quality depth-based FR are FRGC v2 [18] and BU3D-FE [19], and the databases often used in RGB-D FR are Lock3DFace [20], CurtinFace [12], Eurecom [13] and so on. Regarding the former and taking the FRGC v2 as an example, it consists of 4007 3D facial scans of 466 subjects acquired by using a laser 3D scanner, i.e., Konica Minolta Vivid 910. These 3D scans have relatively high resolution and precision. Specifically, their resolution ranges from 50 K to 170 K, and their precision is about 0.1 mm.

Moreover, as mentioned in Section 1, we proposed a database named Multi-Dim [21] in 2017 which contains 124 subjects and in total 124 3D high-quality 3D face shapes. To study boosting face recognition by 3D reconstruction, that database also collected 124 high definition 2D photos, 4305 still face images of acted poses and expressions, and 496 surveillance video clips of varying illuminations and spontaneous poses and expressions.

The Lock3DFace is the largest public database in RGB-D face recognition, which captures face data by using the low-cost RGB-D sensor Kinect II in lab. It contains totally 5711 RGB-D video sequences of 509 Chinese subjects, and the resolution and precision of the obtained 3D face data are 20K and ≥2 mm. We can see it that the quality of two kind of data have a relative gap. There are also some databases containing both RGB and low-quality depth images, such as CurtinFaces [12], Eurecom [13] and IIIT-D [25]. They are all often used in RGB-D FR research. In addition, another RGB-D databases such as HRRFaceD [26], Biwi [27] and Pandora [28] can be also used in depth-based FR research, though they were originally proposed for pose estimation.

Here, in Table 1, we list the main informations of some databases mentioned above and the latest rank-1 identification performance on them. We omit several databases (i.e., HRRFaceD, BIWI and Pandora) and the reasons are (1) the numbers of subjects are less than 50, and the scales of them are small; (2) the researchers made verification mode on them, and we do not find the identification performance of them.

### 2.2. Methods

#### 2.2.1. High-Quality Depth-Based FR

With the high-quality depth information, the performance is very high, and in this scenario, the 3D shapes are usually used. The [1] used a simulated annealing-based approach (SA) for range image registration with the surface interpenetration measure (SIM), as similarity measure to match two face images, which obtained 99.6% in FRGC v2. In [3], the authors presented an approach for computing a compact and highly discriminant biometric signature for 3D face recognition using linear dimensionality reduction techniques, which accessed 99.3% rank-1 identification accuracy on BU3D-FE.

#### 2.2.2. Low-Quality Depth-Based FR

Low-quality depth-based FR is usually as a part of RGB-D FR, which uses texture and depth images at the same time to do FR. The [15] proposed an approach for RGB-D face recognition that is able to learn complementary features from multiple modalities and common features between different modalities, which had the rank-1 accuracy about 66.0% on Lock3DFace only using depth data. In [12], Li et al. extracted multiple features and fuses them at the feature level, which achieved 72.5% recognition rate only using low-quality depth data. In all of the works, the researchers paid more attention to cross-modality FR than low-quality depth-based FR, and paid more attention to tricks and methods on extractor network than data quality. Meanwhile, the depth images are used in this kind of FR, which is a single-view map of depth 3D shapes [29], therefore these methods were not very robust to pose variation. In addition, there was a work [30] based on traditional method focusing on only depth-based work. The authors proposed a descriptor to depth image especially, and it can increase its capacity to distinguish different depth patterns.

#### 2.2.3. Depth Data Enhancement

There is rare work to enhance depth data quality to improve depth-based FR. The [23] proposed some preprocess method including nose tip detection, face cropping, pose correction and symmetric filling for hole filling and smoothing of the depth images by Kinect, and used sparse coding for RGB-D-based FR. However, the purpose of the method was to solve large pose variation. In [24], Kinect fusion was used to fuse several low-quality 3D shapes to obtain a relatively high-quality shape, but the method depended on camera calibration and needed at least two other sensors, which was usually adopted in depth estimation rather than FR. Meanwhile, the two works improved the data quality in data preprocessing, with which visually the data quality had been enhanced, but it was not definitely useful for preserving more identity information for FR. Therefore, our work is to focus on only low-quality depth-based FR, and aims to improve recognition rate by enhancing the depth data quality including both density and precision as well as preserving identity information.

## 3. Extended-Multi-Dim Database

As aforementioned, there is no large-scale public database containing both high and low-quality depth data, which limits the development of depth-based FR, so we extended a multi-modality face database based on Multi-Dim database, namely the Extended-Multi-Dim database. To the best our knowledge, the database is currently the first public database with color and corresponding depth images captured by RealSense and high-quality 3D face shapes scanned by high-quality 3D scanner. Another motivation in creating this database is to solve cross-modality FR, and it is the largest database for RGB-D FR, which consists of 902 objects. Next, we will in detail introduce the proposed database from acquisition details, data process and statistics.

### 3.1. Acquisition Details

**Figure 2:**
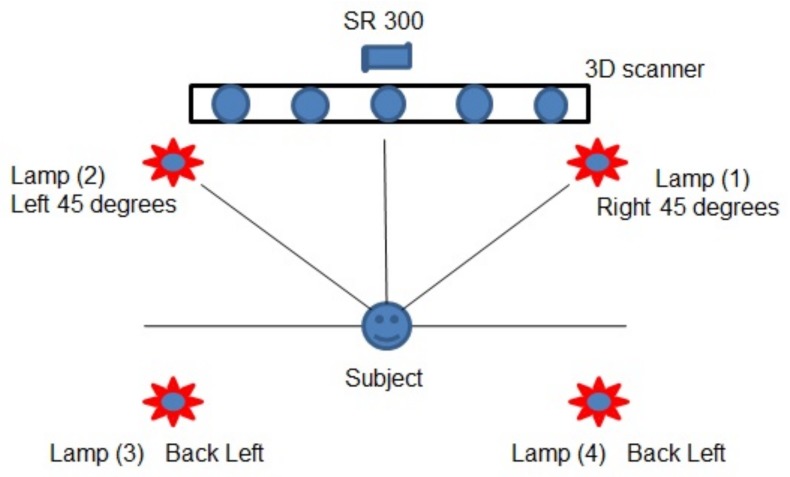
The cameras and lamps were located about 1 m and 1.5 m from the floor respectively and 0.5 m from the subject, while the subject was asked to sit on a chair so that his/her face is about 1 m from the floor.

When capturing RGB and low-quality depth data, the Intel RealSense SR300 was used, and low-quality 3D faces captured by it have a resolution of 45K and a precision of ≥2 mm. Rather than released SDK, we used the tools of a dynamic link library called librealsense [31] to capture RGB and depth videos simultaneously. The RealSense recorded the objects’ videos, and with the librealsense, the videos could be parsed into images when capturing. To align the color faces and their corresponding depth faces, the capturing speed is 22 frames per second, and the resolution of all the images is 960×540. SCU 3D scanner [17] was used to scanning 3D faces, and the 3D faces captured by it have a resolution of 100K and a precision of 0.1 mm. The diagram of data acquisition procedure is shown in Figure 2, where it shows how to record the multi-modal data via Intel RealSence SR300 camera and SCU scanner. The extended database has two versions, which were captured in two different places. The version I consists of 228 subjects, and the Version II has 705 subjects. There are 31 subjects overlapping between the two versions. In previous work [22], when we evaluated how the depth quality influences the depth-based FR, we first extended the Multi-Dim to 228 subjects and captured RGB-D data with RealSense covering three expression variations and yaw direction pose variation. Later, in Version II we further expanded complexity of the pose and expression variations and enlarged the scale of the data set to better simulate a real scene.

To comprehensively evaluate FR methods, especially to simulate complex conditions in the real world, when capturing RGB-D data, volunteers were required to present different expressions, poses under different illumination conditions forming four categories of frontal neutral, expression, pose and illumination. The four parts are introduced in detail respectively in the following:

(1) The illumination variations are shown in Table 2.
(2) The volunteers were scanned in the frontal pose without any expression (referred to as NU for short) for a few seconds in both versions.
(3) The subjects were asked to rotate their heads in yaw direction by $-90^{\circ }$ to $+90^{\circ }$ (referred to as P1 for short) in version I. Apart from these actions, subject’s head was clockwise around the inverse (referred to as P2 for short) in Version II.
(4) In version I, the participants were asked to perform neutral, happy and surprise expressions in the frontal pose, while in Version II, eyebrow lifting, eyes closing, mouth opening, nose wrinkling and teeth barring were asked to be done by volunteers (referred to as FE for short).

**Figure 3:**
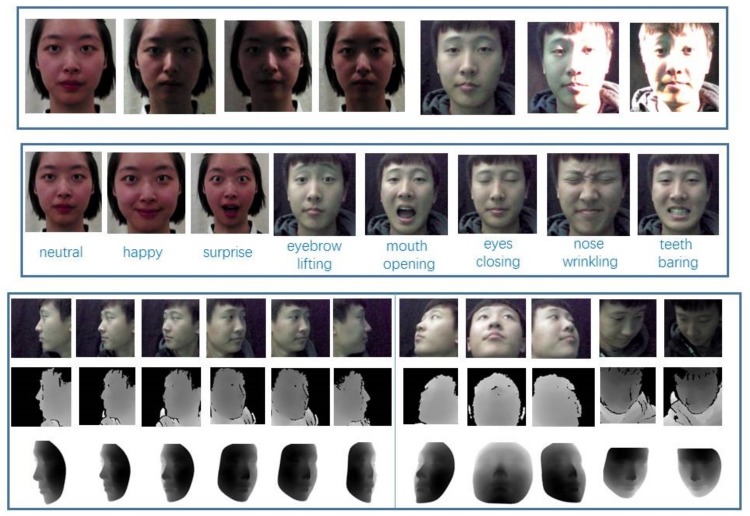
Visual examples in the proposed database. The first line shows the different light conditions where the girl and boy are in version I and II, respectively. The second line shows the different facial expression variations, including 3 expressions of version I and 5 FEs of Version II. The last three lines show the different poses of color, low-quality depth and high-quality depth images, and the first six images belong to P1 while others belong to P2 in each line.

When scanning 3D shapes, the performers only sit still about 0.5 m from the 3D camera under natural light (all lamps are off), no actions were needed. Table 3 displays the overall base information on the database and Figure 3 shows some visual examples.

### 3.2. Data Processing

**Figure 4:**
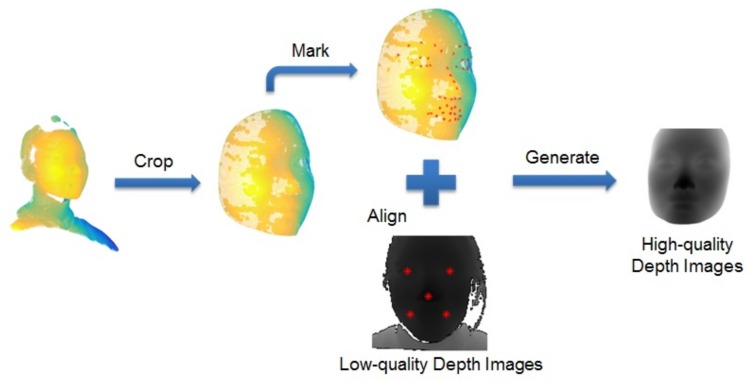
Procedure of generating high-quality depth images.

After the original data are collected, we took some measures to process the data including labeling landmarks, images aligning for FR or other face-based tasks. Regarding the RGB-D data, face and landmarks are hard to be detected with depth images by some open source methods such as MTCNN [32], therefore their aligned color images were either automatically detected by using MTCNN or manually marked (if MTCNN fails). When dealing with 3D shapes, first, we used a commercial application called Geomagic Studio [33] to crop face region manually, then with an open source tool CloudCompare [34], we marked manually 51 landmarks of the cropped shapes whose resolution is between 38K and 89K. Then, we used the 5 landmarks of left and right eye centers, left and right mouth corners, and nose tip of low-quality depth images and corresponding five 3D landmarks to compute the transfer matrix, with which the cropped shapes can be rotated to the requested location. Finally, these rotated faces were projected to 2D planes via weak perspective projection, resulting in high-quality depth images aligned to the low-quality depth images, which created pairs of different quality depth data for training FR models later. The Figure 4 shows the procedure how to use original shapes and low-quality depth images with five landmarks to generate corresponding high-quality depth images. Meanwhile, the Figure 3 shows some examples of aligned high- and low-quality depth images with different pose variations.

### 3.3. Statistics and Protocol

For other researchers expediently using the database and compare the performance, we design a standard experimental protocol for the collected database. Table 3 presents the main statistics of the Extended-Multi-Dim database. In the paper, we focus on how different depth data quality influences the depth-based FR performance and how to improve identification rate by enhancing the depth data quality. Therefore, the whole database can be divided two parts: Training set and Testing set. The former includes pairs of depth images for training FR models, while the latter is for identification (1 to N) FR task, so the Testing set consists of Gallery and Probe. We also care about how depth quality effects the FR performance under different external challenges including pose and face expression variation, so the probe can be divided into four categories: NU, PS1, PS2 and FE. Details are shown below:

(1) Training set: The training data are all from Version II, and except for 31 subjects with Version I, Version II has 674 subjects. We randomly select 430 subjects of the 674 subjects as training sets. In training models, after shuffling training images, the first 20% images are separated into validation sets.
(2) The Testing set are divided into A and B parts, where the remaining 275 subjects in Version II make of the Testing set A and the all data in Version I make of the Testing set B. In Sec V, in different experiments, the specific dividing of galleries and probes can be displayed.
(3) Resolved from original videos and face cropping, there are about 299K, 80K, 318K frames in total for training, validation, and Testing sets, respectively. Owing to the huge amount of data and especially the similarity in joint images, when testing, we select one frame out of every 10 frames in Test set A and every 6 frames in Test set B.

All subjects are Chinese people, and the information of gender statistics are that the ratio of female is 28.1% (64 of 228) while the ratio of male is 72.9% (164 of 228) in version I and that the ratio of female is 43.3% (305 of 705) while the ratio of male is 56.7% (400 of 705) in Version II. In addition, due to the database collecting in the campus, the age of all the subjects is range from 18 to 24 years old.

## 4. Proposed Approaches

The purposes of the work are further to analyze the influence of depth data quality for depth-based FR based on the previous work and meanwhile to improve recognition performance by enhancing the data quality and feature representation. Therefore, we propose three strategies including image-based, feature-based and fusion-based. With the guidance of high-quality of data, we can transfer some knowledge for training a better low-quality depth-based FR model. In this section, we first show our proposed method based on different strategies in detail, and then introduce the backbone models used in the methods.

### 4.1. Image-Based Boosting Strategy

**Figure 5:**
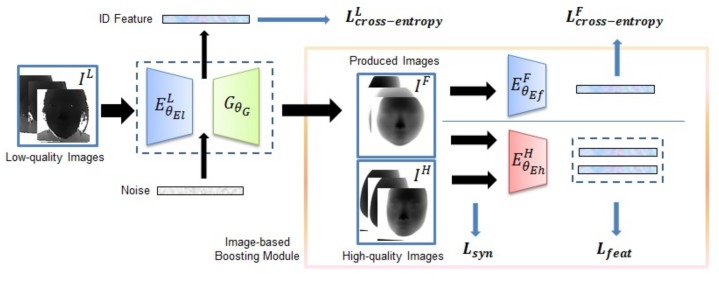
The workflow for image-based strategy. The blue extractors and green generator are needed to be trained and red extractor has been pretrained. The feature represented by ID Feature is finally used for matching. Although the all losses are shown in figure, in different specific methods, the parts of them are used, and details are shown in Section 4.1.

The Figure 5 shows the workflow of the image-based boosting approach. The base purpose of the strategy is to access the identity feature (ID Feature) of low-quality depth image $I^{L}$ through a feature extractor $E_{\theta_{El}}^{L}$, which is a convolution network parameterized by $\theta_{El}$. Generally, ID Feature, the output of $E_{\theta_{El}}^{L}$, is usually used for classification task with the cross-entropy loss $L_{cross-entropy}^{L}$.

To make the ID Feature more discriminative, we simply think that we generate a fake depth face image $I^{F}$ with this ID Feature. If the more similar the produced image and the corresponding high-quality image are, the more discriminative the ID Feature is. So, when training, a generator $G_{\theta_{G}}$ is adopted to ID Feature for production. The generator is a deconvolution network $G_{\theta_{G}}$ to generate a fake image that is parameterized by $\theta_{G}$ with a constraint $L_{syn}$. Also, with the experience from [35], we add a random noise with identity feature to $G_{\theta_{G}}$ , and the noise models facial appearance variations other than identity or data quality.

In addition, we think that if the generated fake images $I^{F}$ also preserve identity information as corresponding ground truth $I^{H}$, the probability distribution of ID Feature is further similar to the one of the high-quality image and the feature is more discriminative. Therefore we conduct two measures: (1) as Equation (3) shows after generating the images, we used the pretrained high-quality depth-based models $E_{\theta_{Eh}}^{H}$ to extract the identity feature of pairs of $I^{F}$ and $I^{H}$, then used loss $L_{feat}$ as a constraint to make the two features similar; (2) from Equation (4), we straightly add another extractor $E_{\theta_{Ef}}^{F}$ and another cross-entropy loss $L_{cross-entropy}^{F}$ after $I^{F}$.

The network’s parameters $\theta_{El}$, $\theta_{G}$ or $\theta_{Ef}$ are optimized by minimizing the aforementioned synthesis loss $L_{syn}$, $L_{cross-entropy}^{L}$ and $L_{feat}$ or $L_{cross-entropy}^{F}$. For a Training set with *N* training pairs of $\left\{I_{n}^{L},I_{n}^{H}\right\}$, the optimization problem can be formulated as follows:

(3) $$\begin{array}{c} \left({\hat{\theta }}_{El},{\hat{\theta }}_{G}\right)=\frac{1}{N}\underset{\theta_{El},\theta_{G}}{argmin}\sum_{n=1}^{N}\{L_{cross-entropy}^{L}\left(E_{\theta_{El}}^{L}\left(I_{n}^{L}\right),y_{n}\right)+\lambda_{1}*L_{syn}\left(G_{\theta_{G}}\left(E_{\theta_{El}}^{L}\left(I_{n}^{L}\right)\right),I_{n}^{H}\right)\\ +\lambda_{2}*L_{feat}\left(E_{\theta_{Eh}}^{H}\left(G_{\theta_{G}}\left(E_{\theta_{El}}^{L}\left(I_{n}^{L}\right)\right)\right),E_{\theta_{Eh}}^{H}\left(I_{n}^{H}\right)\right)\} \end{array}$$

or

(4) $$\begin{array}{c} \left({\hat{\theta }}_{El},{\hat{\theta }}_{G},{\hat{\theta }}_{Ef}\right)=\frac{1}{N}\underset{\theta_{El},\theta_{G},\theta_{Ef}}{argmin}\sum_{n=1}^{N}\{L_{cross-entropy}^{L}\left(E_{\theta_{El}}^{L}\left(I_{n}^{L}\right),y_{n}\right)+\lambda_{1}*L_{syn}\left(G_{\theta_{G}}\left(E_{\theta_{El}}^{L}\left(I_{n}^{L}\right)\right),I_{n}^{H}\right)\\ +\lambda_{3}*L_{cross-entropy}^{F}\left(E_{\theta_{Ef}}^{F}\left(G_{\theta_{G}}\left(E_{\theta_{El}}^{L}\left(I_{n}^{L}\right)\right)\right),y_{n}\right)\} \end{array}$$

where λs are weighting parameters, $L_{syn}$ is defined as L1 loss that jointly constrains a produced image to similar to the high-quality one, and superscript *L*, *H*, *F* represents the low or high-quality images or fake produced images. $L_{feat}$ is Euclidean distance loss (L2 loss). We will postpone the detailed description of all the individual loss functions in Section 5.1.

### 4.2. Feature-Based Boosting Strategy

**Figure 6:**
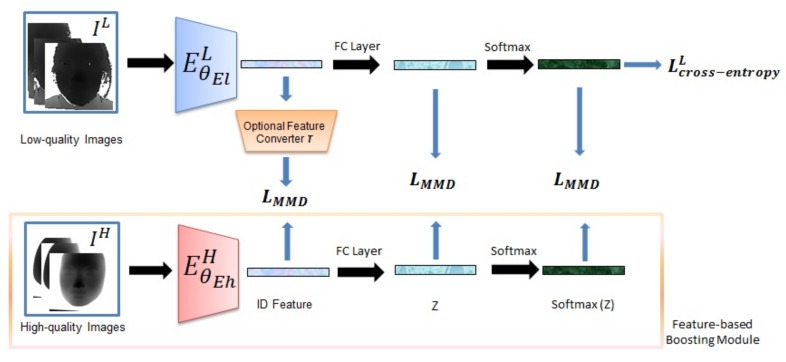
The workflow for feature-based strategy. The blue extractor and orange converter are needed to be trained and red extractor has been pretrained. The feature represented by ID Feature is finally used for FR task. Although the all losses are shown in figure, in different specific methods, the parts of them are used, and details are shown in Section 4.2.

Figure 6 shows the workflow of the feature-based boosting approach. The base purpose of the strategy is same with the one of image-based boosting strategy. Furthermore, in this strategy, we aim to transfer the knowledge of high-quality depth-based extractor $E_{\theta_{Eh}}^{H}$ to learn a corresponding low-quality extractor $E_{\theta_{El}}^{L}$. We expect that the $E_{\theta_{El}}^{L}$ can extract the ID Feature with the similar probability distribution compared with the ones of high-quality images. In this part, inspired by some ideas from the transfer learning [36], we directly and indirectly use constraints to make the two probability distributions similar.

Generally, the last two outputs of a deep FR model or classification model are the logits and ID Feature, and here let us denote the final score output as *Z*. Here, the $E_{\theta_{Eh}}^{H}$ and $E_{\theta_{El}}^{L}$ have the same structure. The $E_{\theta_{Eh}}^{H}$ is first pretrained base on high-quality data. When training the $E_{\theta_{El}}^{L}$, as shown in Figure 6, the input is a pair of images $\left\{I^{H},I^{L}\right\}$, and with the pretrained model, we transfer the knowledge by some losses.

For direct constraints, formulated by Equation (5), we recognize the features from two models as two distributions, and use multi-kernel maximum mean discrepancy (MK-MMD) loss which is often used in many transfer learning works [37] to make the two features similar.

Regarding the indirect constraints, we adopt two methods: (1) formulated by Equation (6), we use MK-MMD loss on margin distribution (*Z*), conditional distribution (softmax(Z)) of the two models as hint to guarantee the features similar; (2) based on feature space transformation, as Equation (7) shows, we transform the ID Feature from low-quality model to the high-quality feature space with a sample converter (T(·)) which consists of two fully connected layers with ELU, then add a L2 loss $L_{feat}$ on two features. Finally, the parameters $\theta_{El}$ is optimized by minimizing an overall loss $L_{overall}$:

(5) $$L_{overall}=L_{cross-entropy}^{L}+\lambda_{4}*L_{MMD}\left({\mathrm{feat}}_{L},{\mathrm{feat}}_{H}\right)$$

or

(6) $$L_{overall}=L_{cross-entropy}^{L}+\lambda_{5}*L_{MMD}\left(Z_{L},Z_{H}\right)+\lambda_{6}*L_{MMD}\left(softmax\left(Z_{L}\right),softmax\left(Z_{H}\right)\right)$$

or

(7) $$L_{overall}=L_{cross-entropy}^{L}+\lambda_{7}*L_{feat}\left(T\left({\mathrm{feat}}_{L}\right),{\mathrm{feat}}_{H}\right)$$

The Equationa (5)–(7) represent the losses for directly and indirectly constraints respectively, where feat represents the ID Feature, λs are weighting parameters, the T(·) is the feature converter and the subscript *L* and *H* represents the vectors from low or high-quality models. We will postpone the detailed description of all the individual loss functions in Section 5.1. Here, MMD is widely used as a distribution distance to measure the discrepancy between two domains. It compares the distributions in the Reproducing Kernel Hilbert Space (RKHS) [38]. The equation for MMD can be formulated as:

(8) $$L_{MMD}\left(x,y\right)=∥\frac{1}{N}\sum_{i=1}^{N}\varphi \left(x^{i}\right)-\frac{1}{M}\sum_{j=1}^{M}\varphi \left(y^{j}\right)∥$$

In the Equation (8), φ(·) is an explicit mapping function. $x^{i}$ and $y^{j}$ represent two samples from distributions of high- and low-quality models. Generally, *N* and *M* are the total numbers of samples, so in our experiments, they are same. By expanding Equation (8), the equation can be reformulated as:

(9) $$L_{MMD}\left(x,y\right)=\frac{1}{N^{2}}\sum_{i=1}^{N}\sum_{i^{\prime }=1}^{N}\kappa \left(x^{i},x^{i}\prime \right)+\frac{1}{M^{2}}\sum_{j=1}^{M}\sum_{j^{\prime }=1}^{M}\kappa \left(y^{j},y^{j}\prime \right)-\frac{2}{NM}\sum_{i=1}^{N}\sum_{j=1}^{M}\kappa \left(x^{i},y^{j}\prime \right)$$

From Equation (9), we can see that MMD loss use kernel method to project the sample vectors into higher dimension. In our experiment, we choose the Gaussian RBF kernel, which is considered to be a universal approximator, with the kernel function as $\kappa \left(x,y\right)=\exp\left(-\frac{{∥x-y∥}^{2}}{2\sigma^{2}}\right)$, where σ is the bandwidth.

### 4.3. Fusion-Based Boosting Strategy

In the part, the main idea is using information of both high-quality image and the feature to guide the low-quality depth-based models training simultaneously. Concretely, we combine some losses of image-based or feature-based methods in the strategy with a simple principle that this combination should improve the FR accuracy relatively obviously. Therefore, according to the results of image-based and feature-based methods, we select some ones with good performance and combine them together.

According to the results, and with a sample purpose that combine the outstanding methods from the two strategies to make the best performance, we finally decide to combine three groups in this part: (1) the methods represented by Equations (4) and (6); (2) The methods represented by Equation (3) and (6); (3) The methods represented by Equations (4) and (7). Here, before adding constraints for two identity features, the normalizations are adopted.

In all combinations, the feature extractor $E_{\theta_{El}}^{L}$ are shared for both image-based and feature-based boosting strategies to gain identity feature for matching, and other methods in image-based or feature-based boosting modules are fused to guide the $E_{\theta_{El}}^{L}$ to extract more discriminative feature.

### 4.4. Backbone Models

In our experiments, the base network has two functions: (1) The performance of the models trained directly severs as the baseline for the models trained based on another strategies, (2) This network structure will be as different part to be assembled for the overall structures of proposed methods.

Here, two deep face recognition models, CASIA-Net [39] and Resnet [40], are considered to be base networks. All are relatively light-weight models. This enables us not only to assemble overall structures together easily but also to train them from scratch by using relatively small data sets of facial depth images that are available to us. Therefore, we do not employ complex or very deep models such as VGG [41] and GoogleNet [42].

For CASIA-Net, motivated by [35], we add batch normalization [43] and exponential linear unit [44] after each convolutional layer. The input image size is changed from 100×100 to 128×128, and the 320-dimensional output of Pool5 layer is taken as the extracted feature.

For Resnet, we employ Resnet-18 as defined in [40]. Its input image size is changed from 256×256 to 128×128, and we also add batch normalization and exponential linear unit after each convolutional layer. Finally, the 512-dimensional output of FC1 is taken as the extracted feature.

In the experiments, either of the two networks is used as feature extractor. Meanwhile the symmetric structure of CASIA-Net is employed as the generator in all image-based schemes. Table 4 shows the specific structure of the networks. For all the deep models, cosine similarity is employed to measure the similarity between the extracted features of different facial depth images.

## 5. Experiments and Results

### 5.1. Experiment Setting

#### 5.1.1. Testing Data Organization

As in Section 3.3, the Extended-Multi-Dim database is divided into training and Testing data sets. In Testing data set which consists of A and B parts, to explore how quality of depth data influence the FR performance under external variations, we select one frontal neutral face image of each subjects in set A as gallery named gallery-A and the remaining images belong to probe. According to the variations, the probe can be divided as probe-A-NU, probe-A-FE, probe-A-PS1 and probe-A-PS2, which consist of the frontal neutral face images, the face images with face expression, the face images with pose variation in yaw direction and the face images with pose variation in all directions.

Regarding set B, straightforward, the gallery-B has the one frontal neutral face image of each subject, and the other images belong to probe-B-all. The details of data organization are shown in Table 5.

#### 5.1.2. Implementation Details

We implement all deep models on TensorFlow [45]. When training them from scratch, the model is initialized by a zero-centered normal distribution with a standard deviation of 0.02, and optimized by using the Adam optimizer [46]. The learning rate is first set as 1e−2 and updated to 1e−4 when the training is saturated. All batch sizes are 64, and we train all models for 10 epochs, and save the models whose accuracy on validation subset is the highest (mostly the accuracy is as high as to 100%). Both baseline models and pretrained high-quality-based models adopt the same setting. Additionally, in image-based strategy, the hyper-parameters $\lambda_{1}$, $\lambda_{2}$, $\lambda_{3}$ are set as 50,1,1 respectively and in feature-based strategy, the hyper-parameters $\lambda_{4}$, $\lambda_{5}$, $\lambda_{6}$, $\lambda_{7}$ are set to 10,10,10,10 and the bandwidth σ is [1,2,5,10,20,40]. We evaluate the depth-based face recognition performance of these deep models in identification mode, and compare their rank-1 identification rates.

Additionally, in image-based strategy, the hyper-parameters $\lambda_{1}$, $\lambda_{2}$, $\lambda_{3}$ are set as 50,1,1 respectively and in feature-based strategy, the hyper-parameters $\lambda_{4}$, $\lambda_{5}$, $\lambda_{6}$, $\lambda_{7}$ are set to 10,10,10,10 and the bandwidth σ is [1,2,5,10,20,40]. We evaluate the depth-based face recognition performance of these deep models in identification mode, and compare their rank-1 identification rates.

### 5.2. Evaluation of Proposed Approaches

#### 5.2.1. The Performance on the Base Models

Here, we train two base networks (CASIA-Net and Resnet-18) on low- and high-quality training data and the high-quality models will be used when training image/feature-based deep models. With the performance shown in Table 6, we observe that while the model structures are different, the performance on models trained on high-quality data is much more outstanding than ones trained on low-quality data. In addition, we notice an interesting phenomenon that the performance gaps between two models trained on two quality data are different under different external variations, i.e., the identification rate gaps on probe-A-NU are much smaller than gaps on probe-A-PS1/PS2 (e.g., for CASIA models, the gaps in NU, PS1, PS2 are 11.7%, 48.0% and 57.4%). We think the main cause is that while there are some self-occlusion in face images with pose variation, the identity information preserved in high-quality images is more accurate than the low-quality images, and with only part of the accurate identity information, the deep model can still extract the effective and discriminative feature for FR. These all show that the data quality is indeed a significant factor influencing the recognition rates for depth-based FR, and demonstrate that it is reasonable to improve FR performance by enhancing depth data quality. Maybe enhancing the data quality can make the data more robust to environmental variations.

#### 5.2.2. The Performance of the Image-based Boosting Models

In the image-based strategy, when we train an extractor for low-quality images, in the rear of the extractor we add a generator and we use L1 loss $L_{syn}$ as a constraint between produced one and ground truth. In addition, to make the produced image discriminative, we adopt two means: directly adding another classifier or comparing the two features extracted from produced one and ground truth by well-trained high-quality deep model. For the later, we use L2 loss for features or normalized features to make a constraint. For feature normalizing, the features are normalized as x/∥x∥ to make them have the same scale, and with [47], the optimization of normalized feature L2 loss $L_{feat_{N}orm}$ becomes consistent with cosine similarity compared with the feature L2 loss $L_{feat}$, which can make an performance improvement. In all evaluations, only the identity features extracted from low-quality images are used to match. Table 7 shows the results of proposed image-based methods, which demonstrates the feasibility of the strategy that when training, the later constraint for similarity of ground truth and produced image from low-quality images can boost the front extractor to gain a more discriminative feature. Additionally, the more identity information the produced images preserve, the more effective this method is. However, we observe that although the performance on data with pose variation has some improvement, there is also a huge gap compared with the performance of high-quality models. Probably, this is because the low-quality depth images are not so accurate originally and under pose variation, so much identity is lost. Therefore, while the high-quality information gives good guidance, it is hard to make up the loss from both internal noisy and external identity lacking.

#### 5.2.3. The Performance of the Feature-Based Boosting Models

In the feature-based strategy, we define the strategy as learning how to make the two probability distributions similar. Here, we add multi-kernel MMD loss on identity feature directly or output of marginal and conditional distributions, which aims to indirectly make the two kind of identity feature distributions approximate. Also, we use the feature transformation to make the two features in the same feature space.

As shown in Table 8, the results show that the guidance of high-quality feature, is indeed useful for improving FR accuracy. However, directly adding the constraint on the high and low-quality features slightly hurts the accuracy, which may be caused that roughly making two features similar may loss some identity information on the produce where the low-quality feature is trying to modeling the distribution of the high-quality one. Meanwhile, with relatively soft methods, it makes a balance between preserving identity information and modeling the high-quality feature distribution. In addition, in both image/feature-based methods, feature normalization makes some sense and especially under pose variations, using feature normalization is much effective.

#### 5.2.4. The Performance of the Fusion Models

As Table 9 shows, there are general improvement on performance compared with the baseline, but some combinations have negative effect. We make the positive results bold and underline the negative results in Table 9. Notice that the negative combinations are not regular, and a possible cause is that simply adding numbers of constraints with different feature extractors or converter, the parameters are increased sharply. When training such a network from scratch, the models have some risk to be confused in optimization, i.e., it is hard to make a balance between different tasks represented by different losses. Certainly, the positive combinations make an obvious improvement, such as about 9% on CASIA-Net model.

#### 5.2.5. The Experiment Analysis

**Figure 7:**
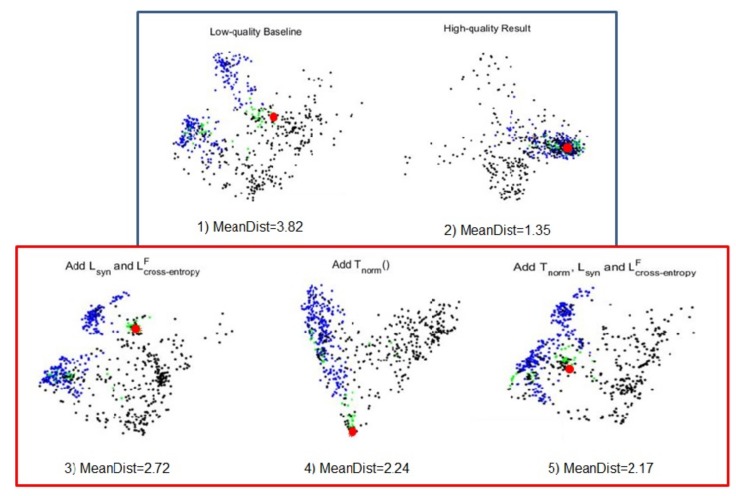
The distribution of the first two dimensions from PCA projections of the same person’s features that are extracted from the proposed methods, where the red point means sample of gallery, the green, blue, black points mean samples of NU, FE, PS in probe, and MeanDist means the Euclidean distance between all features of probe and the one of gallery. The figures in the blue box show the features extracted from low- and high-quality images without any strategies. The features extracted from low-quality images with one of methods on image-based, feature-based and fusion boosting strategies are shown in red box.

The experimental results of all strategies demonstrate that it is feasible to improve depth FR performance by enhancing the depth data quality. In other words, with the methods of three strategies, a more discriminative feature can be acquired under the guidance of the information of high-quality images. In this part, the distributions of the first two dimensions from Principal Component Analysis (PCA) projections of the object’s features that are extracted from the nine models based on CASIA-Net from three strategies and are shown in Figure 7. In all figures, the huge red point means the feature from gallery, and the other green, blue, and black points represent the features from NU, FE and PS of probe. Meanwhile, the mean Euclidean distance between all features of probe and the feature of gallery is displayed. This group of figures straightly show with the proposed methods, intra-class distance is shortened, which means the proposed methods indeed help extractor to acquire more discriminative features.

We believe Figure 7 can relatively explain the results of proposed methods. These methods can effectively deal with the challenge from expression variation, because in Figure 3, Figure 4 and Figure 5 the samples’ features of FE are aggregated and parts of them are close to the one of gallery compared with the first figure. However, even in high-quality result, though the mean discrepancy is small, the pose variation still causes the difference. Therefore, in most methods, while parts of samples in PS are close to the gallery’s and overall discrepancy is decreased, the challenge from pose variation is not solved well by enhancing data quality.

## 6. Conclusions

This paper focuses on using low-quality depth data in face recognition, and we believe that with the knowledge from high-quality data, there will be an improvement on performance of low-quality depth-based models. For the purpose, we collect the first and largest database Extended-Multi-Dim, which includes color, depth images and 3D point clouds of each object at present. Based on this database together with the observations from our previous evaluation, we propose three strategies to use both feature and image information from high-quality data when training a deep model for low-quality depth-based FR. We set a standard protocol of the collected database, based on which we conduct extensive experiments. The results further demonstrate the feasibility of improving depth FR performance by enhancing the depth data quality. Finally, we believe our Extended-Multi-Dim database with the standard protocol will help other researchers, and meanwhile the experimental results and analyses may provide useful clues for camera and sensor manufactures. 

## Figures and Tables

**Table 1:** Benchmark databases and state-of-the-art recognition accuracy on them when using depth images only, RGB images only, or both depth and RGB images.

Databases	No. of Subjects	Devices	Resolution	Precision (mm)	Rank-1 Identification Rate Using		
					Depth	RGB	Depth+RGB
FRGC v2 [18]	466	Vivid 910	60K	0.1	99.6% [1]	–	–
BU-3DFE [19]	100	3dMD	8K	0.2	99.3% [3]	–	–
Lock3DFace [20]	509	Kinect II	20K	≥2	66.0% [15]	92.5% [15]	93.2% [15]
RGBD-W [22]	2239	RealSense	45K	≥2	64.0%	94.7%	–
IIIT-D [25]	106	Kinect I	13K	2–4 [9]	26.8% [11]	99.0% [15]	98.7% [11]
CurtinFaces [12]	52	Kinect I	13K	2–4	72.5% [12]	87.0% [12]	91.3% [12]
Eurecom [13]	52	Kinect I	13K	2–4	69.7% [13]	94.6% [13]	96.3% [15]

**Table 2:** The illumination variations for Extended-Multi-Dim database. The L represents the lamp in Figure 2, and there are four and three illumination variations in version I and Version II, respectively.

Variations	01	02	03	04
Version id	(The conditions of each light in each variation.)			
version I	All off.	L2 on.	L1 on	L3 and L4 on.
version II	All off	L2 on.	L1 and L2 on.	–

**Table 3:** Statistical information of the proposed Extended-Multi-Dim database. The numbers of objects, videos, originally resolved images and cropped and sampled images are shown in training and testing data sets.

	Source	# Obj.	# Videos	# Ori. img.	# Crop. Samp. img.
Training	Version II	430	1290	299K	299K
Testing A	Version II	275	825	256K	40K
Testing B	Version I	228	912	60K	10K

**Table 4:** Network Configuration of Base Model. The format to represent the kernel is ’kernel size, the number of kernels’. For CASIA-Net, downsampling is performed by conv2.1, conv3.1, conv4.1 and conv5.1 with a stride of 2. In Resnet-18, building blocks are shown in brackets, with the numbers of blocks stacked. Here, downsampling is performed by conv3.1, conv4.1, and conv5.1 with a stride of 2.

	Conv1.x	Conv2.x	Conv3.x	Conv4.x	Conv5.x	#feat.
CASIA-Net	3×3.32 3×3.64	3×3.64 3×3.64 3×3.128	3×3.128 3×3.96 3×3.192	3×3.192 3×3.128 3×3.256	3×3.256 3×3.160 3×3.320	320
ResNet-18	7×7,64/2 (max pool.) 3×3/2	$\left[\begin{array}{c} 3\times 3,64\\ 3\times 3,64 \end{array}\right]$ ×2	$\left[\begin{array}{c} 3\times 3,128\\ 3\times 3,128 \end{array}\right]$ ×2	$\left[\begin{array}{c} 3\times 3,256\\ 3\times 3,256 \end{array}\right]$ ×2	$\left[\begin{array}{c} 3\times 3,512\\ 3\times 3,512 \end{array}\right]$ ×2	512

**Table 5:** The standard protocol of low-quality depth-based FR for different scenarios on the Extended-Multi-Dim database. The scales of objects and images and the variations in each part are also shown, where N, I, P, E indicate neutral and frontal, illumination, pose and expression. Notice, $I_{1}$ is natural light and $I_{all}$ contains the all illumination variations shown in Table 2.

Datasets	# Objects.	# Images.	Variations
Gallery-A	275	275	N, $I_{1}$
Probe-A-NU	275	15K	N, $I_{all}$
Probe-A-PS1	275	7K	N, $I_{all}$, $P_{1}$
Probe-A-PS2	275	7K	N, $I_{all}$, $P_{2}$
Probe-A-FE	275	10K	N, $I_{all}$, E
Gallery-B	228	228	N, $I_{1}$
Probe-B	228	10K	N, $I_{all}$, E, $P_{1}$

**Table 6:** Rank-1 identification rates (%) of CASIA-Net and ResNet-18 in face recognition on high-quality and low-quality data sets under variations. Pro. represents Probe.

Network	Quality	Pro.-A-NU	Pro.-A-FE	Pro.-A-PS1	Pro.-A-PS2	Pro.-A-Avg
CASIA-Net	High	99.6	99.2	94.0	87.8	96.6
	Low	87.9	8.1	46.0	30.4	70.3
ResNet-18	High	96.9	94.0	67.7	57.0	84.4
	Low	85.6	75.6	37.7	25.0	65.9

**Table 7:** Rank-1 identification rates (%) of image-based network in face recognition on low-quality data sets under variations. The results in ’Pro.-A-Avg’ are the average rates of specific methods, and the best accuracy is made bold.

$E^{L}$	Methods	Pro.-A-NU	Pro.-A-FE	Pro.-A-PS1	Pro.-A-PS2	Pro.-A-Avg
CASIA-Net	Baseline	87.9	80.1	46.0	30.4	70.3
	$+L_{syn}$	91.5	84.1	51.0	33.0	74.1
	$+L_{syn}+L_{feat}$	92.4	86.1	55.0	36.2	76.1
	$+L_{syn}+L_{feat-Norm}$	93.2	88.3	58.4	38.5	78.0
	$+L_{syn}+L_{Cross-entropy}^{F}$	94.0	89.7	58.9	35.2	79.0
ResNet-18	Baseline	85.6	75.6	37.7	25.0	65.9
	$+L_{syn}$	87.9	76.2	40.1	26.5	67.8
	$+L_{syn}+L_{feat}$	88.2	77.3	41.0	26.5	68.1
	$+L_{syn}+L_{feat-Norm}$	87.7	78.8	41.9	27.3	68.7
	$+L_{syn}+L_{Cross-entropy}^{F}$	86.5	78.0	43.2	28.0	68.2

**Table 8:** Rank-1 identification rates (%) of feature-based network in face recognition on low-quality data sets under variations. The results in ’Pro.-A-Avg’ are the average rates of specific methods, and the best accuracy is made bold.

$E^{L}$	Methods	Pro.-A-NU	Pro.-A-FE	Pro.-A-PS1	Pro.-A-PS2	Pro.-A-Avg
CASIA-Net	Baseline	87.9	80.1	46.0	30.4	70.3
	Equ-(5)	87.5	78.0	42.7	28.4	68.7
	Equ-(6)	91.3	84.1	56.7	37.7	75.6
	Equ-(7)	92.3	87.4	51.7	34.5	75.6
	Equ-(7)${}_{Norm}$	92.0	87.0	55.0	36.0	76.2
ResNet-18	Baseline	85.6	75.6	37.7	25.0	65.9
	Equ-(5)	85.0	75.8	38.3	25.4	66.3
	Equ-(6)	87.4	77.8	40.4	27.4	68.0
	Equ-(7)	86.6	77.4	40.0	26.6	67.0
	Equ-(7)${}_{Norm}$	86.9	77.6	42.0	28.0	68.2

**Table 9:** Rank-1 identification rates (%) of fusion-based network in face recognition on low-quality data sets under variations. The results in ’Pro.-A-Avg’ are the average rates of specific methods in Test set A, and the results of the positive combinations are made bold, but the negative ones are added underline. The last column gives the overall performance in Test set B.

$E^{L}$	Methods	Pro.-A-NU	Pro.-A-FE	Pro.-A-PS1	Pro.-A-PS2	Pro.-A-Avg	Pro.-B
CASIA-Net	Baseline	87.9	80.1	46.0	30.4	70.3	56.7
	Equ-(4) + (6)	94.2	90.7	60.8	42.3	80.0	66.4
	Equ-(4) + ${\left(7\right)}_{Norm}$	94.3	89.7	60.8	41.6	79.6	66.2
	Equ-${\left(7\right)}_{Norm}$+(6)	91.7	85.1	52.3	34.7	$\underline{74.8}$	59.3
ResNet-18	Baseline	85.6	75.6	37.7	25.0	65.9	47.5
	Equ-(4) + (6)	86.6	76.4	41.3	28.0	$\underline{67.5}$	50.8
	Equ-(4) + ${\left(7\right)}_{Norm}$	88.1	78.5	43.6	28.7	69.3	54.2
	Equ-${\left(3\right)}_{Norm}$+(6)	87.5	76.3	39.5	26.5	$\underline{67.4}$	49.0
